# Relationships between Strength and Step Frequency with Fatigue Index in Repeated Sprint Ability

**DOI:** 10.3390/ijerph19010196

**Published:** 2021-12-24

**Authors:** Pablo González-Frutos, Millán Aguilar-Navarro, Esther Morencos, Javier Mallo, Santiago Veiga

**Affiliations:** 1 Faculty of Health Sciences, Universidad Francisco de Vitoria, 28223 Madrid, Spain; millan.aguilar@ufv.es (M.A.-N.); esther.morencos@ufv.es (E.M.); 2 Sports Department, Universidad Politécnica de Madrid, 28040 Madrid, Spain; javier.mallo@upm.es (J.M.); santiago.veiga@upm.es (S.V.)

**Keywords:** sport performance analysis, training methods, team sports, monitoring and evaluation of training

## Abstract

Force−velocity profile (FVP) and repeated-sprint ability (RSA) tests are indicators of physical capacities in most team sport players. The purpose of this study was to examine the stride kinematics during a repeated-sprint ability (RSA) test and to analyze the relationship between Bosco’s force−velocity profile (FVP) and RSA performance in elite female field hockey players. Thirteen elite-female players performed both RSA (six 30 m maximal sprints) and jumping (CMJ weighted and body weight) tests. Sprinting time fatigue indexes during a 30 m RSA test were correlated with step frequency fatigue indexes (r > 0.7; *p* < 0.01). CMJ50 showed a large relationship with sprint time fatigue indexes. FV50 showed a very large relationship with sprint time fatigue indexes (r > 0.7; *p* < 0.01), and a large relationship with the step frequency fatigue indexes (r > 0.5; *p* < 0.05). This study highlighted two possible ways to improve fatigue indexes in RSA, with the aim of maximizing the distances covered at high-intensities during the matches: (a) strength training and (b) focusing on step frequency during speed training.

## 1. Introduction

Most team sports are characterized by an intermittent activity where periods of short high-intensity efforts are interchanged with periods of active and passive recovery [1,2,3,4,5,6,7,8]. The total sprint distance covered by players in these disciplines is an important performance factor, as noted in field hockey [1], Australian Football [2], and soccer [3]. Therefore, repeated-sprint ability (RSA) tests have largely been used as an important intermittent sport performance index [4,5], and to track the effectiveness of training programs [6]. Moreover, the ability to perform repeated sprint bouts with a short recovery time between them has been reported to be relevant for field hockey performance and is worth being evaluated and trained [7,8].

From a physiological perspective, RSA is a complex quality that is essential in order to obtain maximal sprint speed and, at the same time, to achieve the oxidative capacity needed for phosphocreatine recovery and hydrogen buffering [9]. Several physical capacities play a determinant role in RSA. For example, lower-limb strength and power provide acceleration and maximal speed during the first repetitions of multiple sprints [10]. This highlights the important of a well-developed neuromuscular system, which for allows a better activation of the motor units [11]. On the other hand, stride kinematics is also a performance determinant in sprinting ability. Specifically, it has been demonstrated that a higher step frequency through a shorter support time can optimize 60 m sprints [12], and that training at a faster step cadence may improve running economy in female distance runners [13]. Additionally, a previous study reported changes in stride kinematics during RSA tests performed on a treadmill, with the step frequency decreasing during the five repetitions of 5 s sprints [14]. This finding has also been described to occur during RSA field tests [15]. However, there is no further information about the evolution of stride kinematics during RSA field tests.

The strength and power values of athletes are critical indicators of sprinting performance, which, at the same time, is the base of RSA [16]. One of the best indicators of lower limb neuromuscular fatigue is CMJ performance, which has been related to sprint ability [17,18] and has been employed within the Bosco Index to identify the force−velocity characteristics of athletes [19,20]. CMJ performance has been used as a tool to individualize training loads during sprinting sessions, providing information about the mechanical and physiological response of athletes, instead of employing a standard fixed number of sprints for all players [21]. In addition, it has been recently reported that CMJ performance is related to specific actions in intermittent-like sports such as field hockey [22]. Nevertheless, no information about the role of force−velocity profile (FVP) on RSA performance has been previously examined.

Therefore, the aim of this study was to examine the stride kinematics during an RSA test, and to analyze the relationship between Bosco´s FVP and RSA performance in elite female field hockey players.

## 2. Materials and Methods

**Figure 1:**
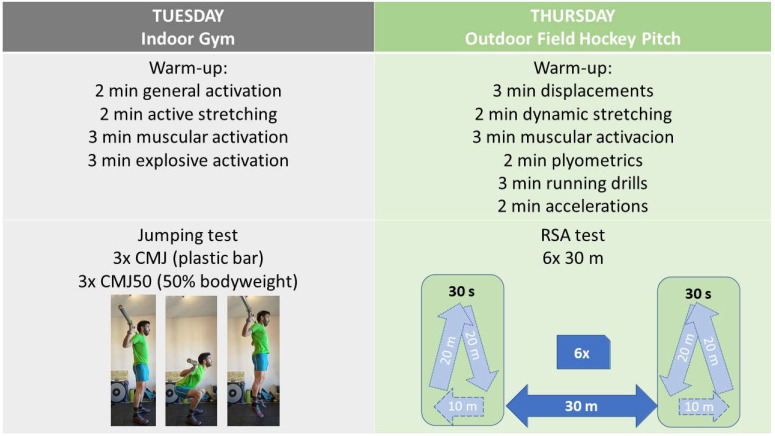
Jumping and RSA test protocols.

Thirteen elite-female field hockey players (age 24.9 ± 5.6 years, body height 1.67 ± 0.04 m, and body weight 58.7 ± 3.7 kg) participated in this study. The study was carried out in the ninth week of the 2018/2019 season. The study was approved by the Local University Ethics Committee, and participants were fully informed about the protocol of the study and gave their informed consent according to the Declaration of Helsinki. The team staff was involved in the design and supervision of the study, so the research protocol was part of the regular training routine. All test sessions were conducted at the same time of day and during the physical training part of the session that preceded the field hockey training. Specifically, the jumping tests were performed in Tuesday’s session (regular strength session), whereas the RSA test was performed in Thursday’s session (regular speed session), both with their respective warm-ups (Figure 1). 

### 2.1. RSA Test

Before testing, all the participants performed a regular, specific, and standardized 15 min warm-up comprising 3 min of jogging and displacements using different movement and orientation patterns, 2 min of dynamic light stretching, 3 min of basic muscular activation, 2 min of plyometrics, 3 min of running drills, and 2 min of short distance accelerations. The RSA protocol consisted of six 30 m maximal sprints interspersed with 30 s of active recovery periods, where players decelerated during 10 m and jogged 40 m to position themselves for a new start [23]. The sprint times were measured using electronic photocells (Microgate, Bolzano, Italy), which were adjusted according to the height of the players and were placed at 0, 10, 20, and 30 m. Each sprint was initiated from a standing position with their foot 1 m behind the first timing gate. One fixed video camera, EX-ZR800 (Casio Computer Co., Tokio, Japan), located in a lateral view and operating at 60 Hz (shutter speed: 1/1000, 1920×1080 px), recorded the 30 m, similar to previous studies [24,25]. The step frequency (SF = number of steps/time of the number of steps) and step length (SL = sprint distance/sprint time/step frequency) were determined using a video analysis of the test. The RSA performance was assessed using five scores for each variable (sprint time, step frequency, and step length): the best, mean, and worst of the six repetitions, and two fatigue indexes, calculated as a percentage decrement from the best value (Fmean = 100 − (mean/best*100); Fworst = 100 − (worst/best*100)) [26].

### 2.2. Jumping Test

Before the test, all the participants performed a regular, specific, and standardized 10 min warm-up comprising 2 min of general activation, 2 min of light active stretching, 3 min of basic bodyweight muscular activation (10 repetitions of lunges, squats, hip thrusts, and single leg dead lifts), and 3 min of explosive activation (six repetitions of squat jumps, CMJ, and drop jumps). The CMJ and a CMJ50 (CMJ with external loads equivalent to 50% of the players´ bodyweight) tests were performed on a contact platform (Chronojump-BoscoSystem, Barcelona, Spain). Jumping height was determined based on the flight time using Chronojump software (Chronojump-BoscoSystem, Barcelona, Spain). The best of three attempts was selected for each jump. As the players held down a bar on their shoulders during the CMJ50, they were also instructed to hold down a plastic bar during the CMJ to mimic the jumping execution. Participants were instructed to start from a standing position; perform a rapid flexion–extension of the legs with a minimum pause between the eccentric and concentric phase of the muscle contraction; bend their knees to a freely chosen angle; perform a maximal jump keeping their body vertical throughout the jump, avoiding undue lateral and frontal movements; and to land with knees fully extended [27,28]. The FV50 was calculated using Bosco´s Index (FVP50 = CMJ50/CMJ*100) [19,20].

### 2.3. Statistical Analysis

All of the results are expressed as the mean and standard deviation (SD). A repeated measures analysis of variance was used to compare the sprint times, step frequency, and step length across the number of repetitions (1 to 6) and section (0–10 m, 10–20 m, and 20–30 m. Post hoc tests were used to determine the statistical effects (*p* < 0.05) between factors using Bonferroni corrections, and were interpreted using effect sizes (ηp2) with 0.2, 0.5, and 0.8 threshold values for small, medium, and large effects [29]. Pearson correlation coefficients were used to relate the PFV parameters with the RSA kinematic parameters, with 0.1, 0.3, 0.5, 0.7, and 0.9 being the threshold values that represented small, moderate, large, very large, and nearly perfect correlations, respectively [30]. Statistical analyses were performed using the IBM Statistical Package for Social Sciences Statistics, version 22.0 (IBM Inc., Armonk, NY, USA).

## 3. Results

### 3.1. Repetitions and Sections Analysis

**Figure 2:**
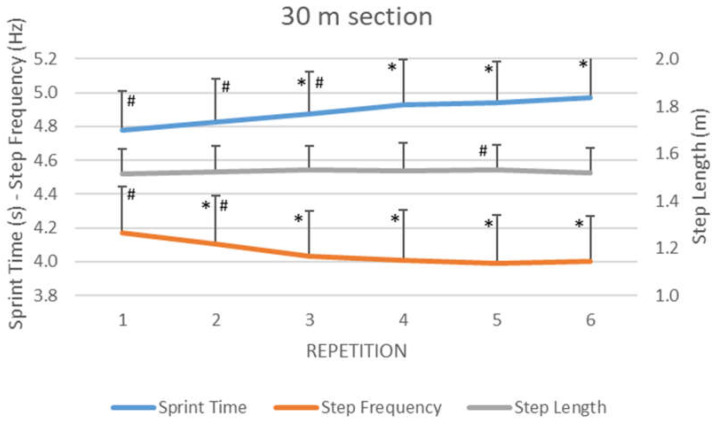
Evolution of step kinematics during RSA in elite female field hockey players. (*) Different from the first repetition at *p* < 0.05; (#) different from the last repetition at *p* < 0.05.

The evolution during the six repetitions of the RSA 30-m sprint test (Figure 2) showed medium differences between repetitions in sprint time (F_3.65_ = 19.73, *p* < 0.001, η2 = 0.62) and step frequency (F_2.70_ = 24.40, *p* < 0.001, η2 = 0.67), and trivial differences in step length (F_4.57_ = 2.88, *p* = 0.026, η2 = 0.19). Pair-wise comparisons in the sprint time and step frequency revealed multiple statistical differences, whereas, for the step length, differences were only found between the last and the fifth repetition (*p* = 0.03). When comparing the first and the last repetition (Table 1), the sprint time increased by 4.1% (*p* < 0.001), whereas step frequency decreased by 4.0% (*p* < 0.001) and the step length remained constant. 

**Figure 3:**
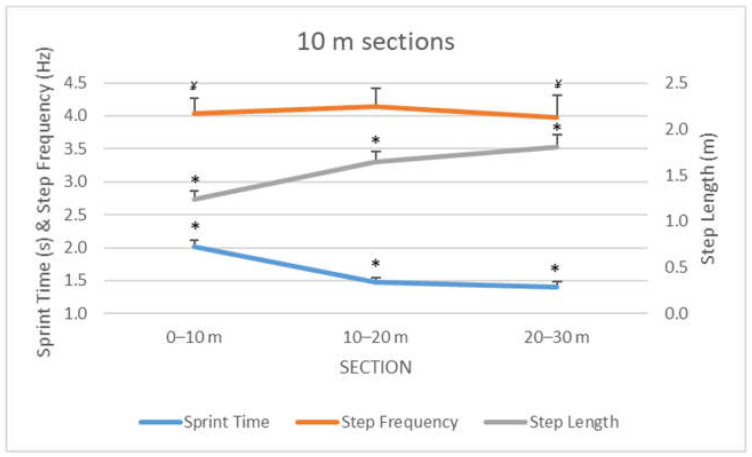
Evolution of step kinematics during the 10 m sections for the RSA test in elite female field hockey players. (*) Different from the rest of sections at *p* < 0.05; (¥) Different from the second section at *p* < 0.05.

The analysis between the sections (0–10 m, 10–20 m, and 20–30 m) showed large differences in sprint time (F_1.39_ = 1198.56, *p* < 0.001, η2 = 0.99) and step length (F_2.24_ = 1039.43, *p* < 0.001, η2 = 0.99), and moderate differences in step frequency (F_1.5_ = 15.65, *p* < 0.001, η2 = 0.57). Pairwise comparisons (Figure 3) showed differences (*p* < 0.001) between all the sections for sprint time and step length, while step frequency showed higher values in the 10–20 m section.

### 3.2. Sprint Time, Step Frequency, and Step Length

The correlation analysis (Table 2) within each variable (sprint time, step frequency, and step length) showed nearly perfect (r > 0.9; *p* < 0.001) relationships between best, mean, and worst 30 m times. In addition, FImean and FIworst had nearly perfect (r > 0.9; *p* < 0.001) or very large (r > 0.7; *p* < 0.001) relationships within each variable (sprint time, step frequency, and step length). However, no relationship was shown between the fatigue indexes with the best, mean, or worst 30 m times in each variable (sprint time, step frequency, and step length).

The correlational analysis between the sprint time, step frequency, and step length (Table 3) showed very large relationships for both the fatigue index between sprint time and step frequency (r > 0.7; *p* < 0.01), and for best, mean, and worst between frequency and step length (r < −0.7; *p* < 0.01). No relationships were found between step length and sprint time.

### 3.3. Jumping Test and Relationships with RSA Performance

**Figure 4:**
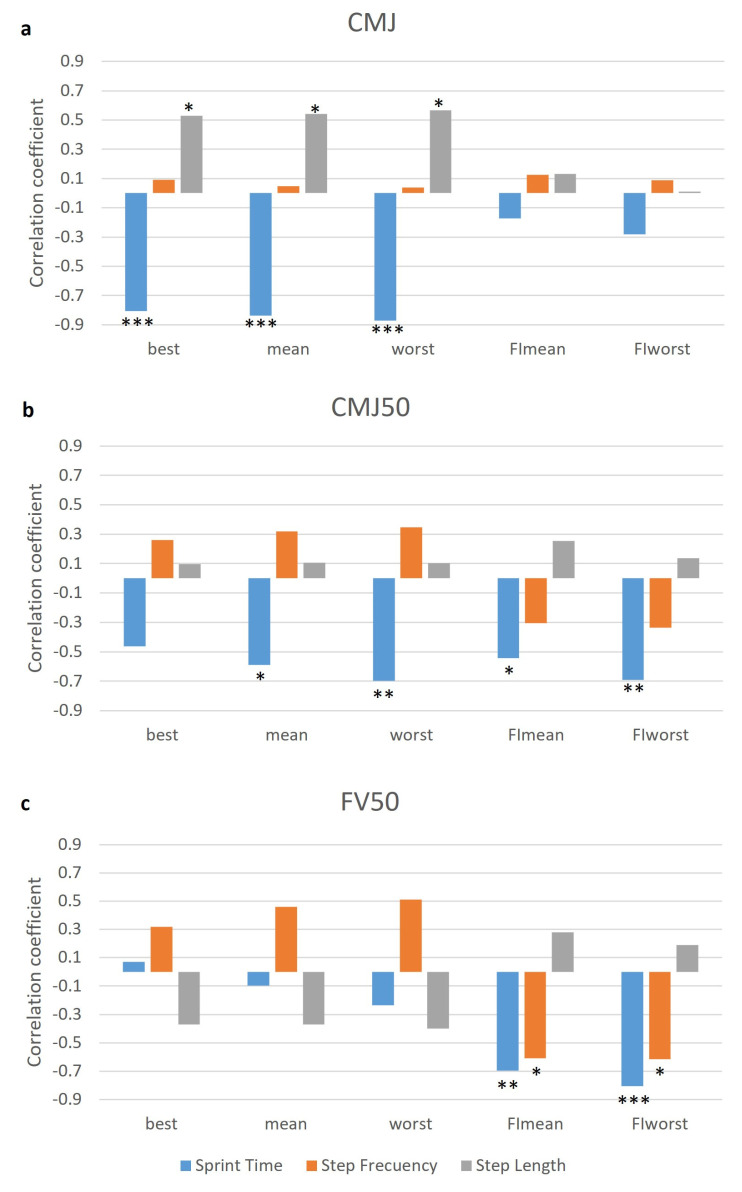
Relationships (r) between (**a**) CMJ, (**b**) CMJ50, and (**c**) FV50 and the best, mean, worst, FImean, and FIworst parameters of step kinematics during the RSA test. Statistical correlation at *p* < 0.05 (*), *p* < 0.01 (**) or *p* < 0.001 (***).

The CMJ parameters (29.8 ± 3.7 cm) showed a very large relationship with the best, mean, and worst sprint time, and a large relationship with the best, mean, and worst step length (Figure 4a). Additionally, the CMJ50 parameters (15.06 ± 3.1 cm) showed a large relationship with the mean, worst, FImean, and FIworst sprint time (Figure 4b). Finally, the FV50 parameters (52.2 ± 7.2) showed a very large relationship with the FImean and FIworst sprint time, and a large relationship with the FImean and FIworst step frequency (Figure 4c).

## 4. Discussion

Despite the reported importance of RSA on intermittent-like sport disciplines, the role of RSA performance determinants such as running kinematics and the force−velocity profile are still unclear. Therefore, the aim of the present research was to examine the stride kinematics during an RSA test, and to analyze the relationships between Bosco FVP and RSA performance in elite female field hockey players. Fatigue indexes during a 30 m RSA test were correlated to CMJ50, FVP50 values and fatigue index of step frequency, but not to CMJ. 

### 4.1. Repetitions and Sections Analysis during RSA Test

The 30 m sprinting times obtained by elite female field hockey players during the present study (average values below 5 s in all six repetitions) were faster than those recorded in female football players (greater than 5 s in seven repetitions) [31]. This confirms the level of the participants in the present research, and ensures that the test was performed under conditions of maximum intensity. Sprint times increased progressively across all repetitions of the RSA test (Figure 2 and Table 1), and significant differences were found between the first sprint and the last four repetitions, in line with previous studies [23,31]. In relation to the step kinematics, the step frequency values (average team value equal or greater than 4Hz in the six repetitions) were higher than those recorded in female football players (equal or lower than 4 Hz in seven repetitions) performed under very similar conditions [31]. However, the step length values were similar (average team value between 1.50 and 1.55 m in all repetitions) to those measured in female football players [31]. The differences found between the first and last repetition were similar to those reported in step kinematics during five 5 s treadmill sprints with 25 s of recovery between them [14].

Step length did not seem to be an important parameter in the RSA test in view of the scarce differences (Table 1), as only small variations were observed between the fifth and sixth repetition (Figure 2). However, the step frequency showed a large decrease from the second repetition onwards, which might suggest that the increment in the total sprinting time could be associated with a decrease in the step frequency, whereas the step length remained stable [31]. This finding could encourage coaches to control step frequency as a method to optimize sprint endurance rather than focusing on a greater step length. In this line, maintaining short ground contact times [12,17,31] could help athletes to increase their step frequency. Another interesting aspect that emerges from the current analysis is how athletes manage fatigue during RSA tests. Differences in step kinematics between repetitions indicate that the athletes presented a better technical execution during the initial repetitions, but there is a fatigue effect from the fourth to the sixth repetition. Consequently, training sets of up to three repetitions could be prescribed to optimize sprint time and step frequency values. On the other hand, training sets of more than three repetitions could be prescribed to improve endurance. These ideas to organize the number of repetitions during training are consistent with previous suggestions in the literature [7]. 

Within the 30 m sprint distance, the step frequency presented small differences between the three 10 m sections (Figure 3), as previously observed in other investigations [14,31,32]. Elite athletes may have an optimum control of their step frequency at a different range of velocities and step lengths, as there were greater differences in these variables in the three 10-m sections (η2 = 0.99). Furthermore, the development of step frequency fatigue would be reflected in efforts between 10 and 30 m.

### 4.2. Sprint Time, Step Frequency and Step Length

Based on the correlations obtained in Table 1, it could be observed that fatigue indexes provide further information than examining the best, average, and worst parameters in isolation, indicating the importance and usefulness of calculating fatigue indexes as RSA performance indicators [33]. Furthermore, the fatigue indexes were not correlated to the best, average, or worst times in any of the step kinematic variables. However, as the sprint time fatigue indexes were only correlated with the step frequency fatigue indexes and not with the step length fatigue indexes, we recommend establishing strategies that will allow athletes to focus and improve frequency control in fatigue situations, in addition to improving top speed [12,17,32,34]. This aspect could be a very useful alternative with younger athletes (U15, U17, and U19) due to their greater fatigue shown in RSA [35].

### 4.3. Jump Test and Relationship with RSA

The correlation found between CMJ and the best, mean, and worst sprinting times is in agreement with other studies that have assessed the efficacy of explosive strength training for the improvement of acceleration and maximum speed in basketball, Australian football, and rugby [17,18,36]. Likewise, previous studies have also reported improvements in maximal velocity, but not in RSA [37], after explosive strength training in young elite male soccer players [11]. For this reason, the most used method to improve RSA fatigue indexes is repeated sprint training itself [11]. Jumping activities with body weight is the load that optimize power output [38], explaining a large part of the performance in the first repetitions [10], even in young players [39].

Based on previous studies, the average CMJ50 jump height obtained in the present research could be associated with a load of 70% of the 1RM in full squat [40]. This value would be classified within a high-load resistance training protocol [41], being sufficient stimulus to maximize adaptations in muscle strength [42]. Positive effects of strength training with greater loads (3x8x75% 1RM) have been found on the RSA fatigue index in futsal players [43]. Similarly, the results of the current investigation with elite female hockey players suggest that training should focus on strength training (CMJ50 and FV50) to obtain improvements in RSA fatigue rates. As the maximal strength (F0) is more compromised than the maximal sprint velocity (V0) over the course of a season in male football players [44], it seems interesting to focus on strength training as a way to improve intra- and inter-session endurance. Moreover, previous studies suggest that the ability to produce a horizontal force at low speed (F0), instead of the maximum speed (V0), is the variable that is altered both before and after the return to sport after an injury [45]. For this reason, it could be assumed that better CMJ50 values can be related not only to better performance due to their relationship with fatigue indices, but also to a lower risk of injury.

According to the results obtained with the methodology used in the present study, it seems that the use of a load of 50% of the total bodyweight provides relevant information for sports performance, due to the correlation between the CMJ50 and FV50 with the RSA variables, specifically with the parameters that are affected by fatigue in the sprint time and even the step frequency.

## 5. Conclusions

In summary, kinematic analysis showed that sprinting time fatigue indexes during 30 m RSA test were scarcely correlated with step frequency fatigue indexes. In addition, the PFV analysis showed that the CMJ50 and FVP50 values were correlated to sprint time and step frequency fatigue indexes during the 30 m RSA test.

### 5.1. Practical Applications

Based on the results obtained in the present investigation, we could highlight two ways to improve fatigue indexes in RSA, with the aim of maximising distances covered at high-intensities during the matches: (a) strength training and (b) focusing on step frequency during speed training. Both strategies have in common that they focus on the neural system, presenting a lower risk of sustaining injuries and a lower interference in specific game training than the traditional approach to RSA training. Additionally, these strategies suppose practical training alternatives that require low economic and temporary training and evaluation costs.

Specifically, the use of CMJ50 to assess the maximum strength levels and the FV50 to describe the force−velocity profile of players is proposed. It is suggested that a 50% bodyweight load is the minimum reference value when performing squat exercises with and without jumping. Furthermore, it is easy for athletes to understand the need to obtain FV50 values greater than 50, although a value close to 60 would be expected as an indicator of a balance in the force−velocity profile [19].

In terms of step frequency training, it could be recommended to reduce step lengths by means of agility ladders, hurdles, or other types of materials that focus the attention on step frequency. In this way, focusing on the stride frequency may avoid increased risk of injury situations (as they demand a lower peak velocity and a smaller ROM in hamstrings). Additionally, the use of plyometric exercises with short contact times, proper body alignment and timing with the arms could also be recommended to master step frequency. It is also worth highlighting the psychological implication (strengthening attention span, avoiding monotony, and decreased perception of effort) of focusing the athlete on specific technical aspects during physical training sessions.

### 5.2. Limitations of the Study

The originality of this study relies on being the first study to examine an elite sample of team sports players in their habitual training environment, analyzing the relationship between the force−velocity profile (and not only the CMJ performance) and the kinematic running variables during an RSA activity. Nevertheless, there are some limitations that must be acknowledged. First, the sample of the study is relatively small, so it would be necessary to carry out more studies in larger samples to corroborate the results obtained in the present investigation. Second, this is a descriptive study where the relationships observed do not neccesarily represent causality. Third, only two training loads for the CMJ test were employed, instead of the four loads method extensively reported in the literature. 

## Figures and Tables

**Table 1:** Percentage changes in relation to the first repetition of the stride kinematics during RSA in elite female field hockey players. (*) Different from the first repetition at *p* < 0.05.

Variables	Repetition				
	2	3	4	5	6
Sprint Time	1.0 ± 0.4%	2.1 ± 0.5% *	3.2 ± 0.6% *	3.5 ± 0.8% *	4.1 ± 0.5% *
Step Frequency	−1.6 ± 0.4%	−3.3 ± 0.5% *	−3.9 ± 0.7% *	−4.4 ± 0.8% *	−4.0 ± 0.6% *
Step Length	0.6 ± 0.4%	1.3 ± 0.5%	0.9 ± 0.5%	1.1 ± 0.5%	0.1 ± 0.5%

**Table 2:** Relationships between the best, mean, worst, FImean, and FIworst parameters within the step kinematics (sprint time, step frequency, and step length) during the RSA test in elite female field hockey players. Bold: Different from the first repetition at *p* < 0.05.

Variables		Sprint Time					Step Frequency					Step Length				
Parameters		Best	Mean	Worst	FImean	FIworst	Best	Mean	Worst	FImean	FIworst	Best	Mean	Worst	FImean	FIworst
best	r		**0.973**	**0.928**	−0.060	−0.060		**0.966**	**0.925**	−0.041	−0.163		**0.999**	**0.992**	0.314	0.313
	*p*		**0.000**	**0.000**	0.846	0.846		**0.000**	**0.000**	0.894	0.594		**0.000**	**0.000**	0.297	0.298
mean	r			**0.985**	0.170	0.161			**0.990**	−0.298	−0.409			**0.995**	0.281	0.286
	*p*			**0.000**	0.579	0.599			**0.000**	0.323	0.165			**0.000**	0.353	0.344
worst	r				0.298	0.317				−0.412	−0.526				0.223	0.200
	*p*				0.323	0.292				0.162	0.065				0.465	0.511
FImean	r					**0.951**					**0.979**					**0.856**
	*p*					**0.000**					**0.000**					**0.000**

**Table 3:** Relationships between step frequency parameters (best, mean, worst, FImean, and FIworst) with sprint time and step length parameters (best, mean, worst, FImean, and FIworst) during the RSA test in elite female field hockey players. Bold: Different from the first repetition at *p* < 0.05.

		Sprint Time					Step Length				
Step Frequency		Best	Mean	Worst	FImean	FIworst	Best	Mean	Worst	FImean	FIworst
best	r	−0.352	−0.380	−0.389	−0.131	−0.152	**−0.707**	**−0.707**	**−0.703**	−0.195	−0.242
	*p*	0.238	0.200	0.189	0.670	0.620	**0.009**	**0.010**	**0.014**	0.524	0.426
mean	r	−0.268	−0.343	−0.380	−0.337	−0.343	**−0.752**	**−0.751**	**−0.731**	−0.185	−0.237
	*p*	0.376	0.251	0.201	0.260	0.252	**0.003**	**0.003**	**0.005**	0.545	0.436
worst	r	−0.234	−0.328	−0.377	−0.420	−0.423	**−0.758**	**−0.756**	**−0.738**	−0.185	−0.217
	*p*	0.442	0.273	0.204	0.153	0.149	**0.003**	**0.003**	**0.004**	0.545	0.476
FImean	r	−0.246	−0.060	0.049	**0.814**	**0.767**	0.359	0.357	0.362	0.009	0.048
	*p*	0.417	0.846	0.873	**0.001**	**0.002**	0.228	0.232	0.224	0.976	0.877
FIworst	r	−0.170	0.012	0.119	**0.793**	**0.759**	0.426	0.420	0.424	0.078	0.067
	*p*	0.579	0.969	0.698	**0.001**	**0.003**	0.147	0.153	0.149	0.800	0.827

## Data Availability

The data presented in this study are available on request from the corresponding author. The data are not publicly available due to restrictions privacy.
